# PTFE-based microreactor system for the continuous synthesis of full-visible-spectrum emitting cesium lead halide perovskite nanocrystals

**DOI:** 10.3762/bjnano.8.252

**Published:** 2017-11-28

**Authors:** Chengxi Zhang, Weiling Luan, Yuhang Yin, Fuqian Yang

**Affiliations:** 1 Key Laboratory of Pressure Systems and Safety (MOE), School of Mechanical and Power Engineering, East China University of Science and Technology, Shanghai 200237, P. R. China,; 2 Department of Chemical and Materials Engineering, University of Kentucky, Lexington, KY 40506, USA

**Keywords:** cesium lead halide, microreactor system, nanocrystals, perovskite, photoluminescence

## Abstract

Colloidal perovskite nanocrystals comprised of all inorganic cesium lead halide (CsPbX_3_, X = Cl, Br, I or a mixture thereof) have potential as optical gain materials due to their high luminescence efficiency. In this work, cesium lead halide nanocrystals are continuously synthesized via a microreactor system consisting of poly(tetrafluoroethylene) (PTFE) capillaries. The synthesized nanocrystals possess excellent optical properties, including a full width at half maximum of 19–35 nm, high fluorescence quantum yield of 47.8–90.55%, and photoluminescence emission in the range of 450–700 nm. For the same precursor concentrations, the photoluminescence emission peak generally increases with increasing reaction temperature, revealing a controllable temperature effect on the photoluminescence characteristics of the synthesized nanocrystals. For quantum dots synthesized with a Br/I ratio of 1:3, a slight blue shift was observed for reaction temperatures greater than 100 °C. This PTFE-based microreactor system provides the unique capability of continuously synthesizing high-quality perovskite nanocrystals that emit over the full visible spectrum with applications ranging from displays and optoelectronic devices.

## Introduction

Colloidal semiconductor nanocrystals, also known as quantum dots (QDs), have great potential in a variety of applications, including displays, solar cells, lasers, light-emitting diodes and white-light generation [1–6] due to their size-tunable optical and electrical properties and excellent ability to be processed in solution. Quantum dots have gained much attention due to their promising applications. There are reports on the synthesis of colloidal, hybrid organic–inorganic perovskite QDs (CH_3_NH_3_PbX_3_, X = Cl, Br, I and their mixture thereof) and all inorganic perovskite QDs (CsPbX_3_, X = Cl, Br, I and their mixture thereof) [7–9], which likely can be used as a photosensitive layer for photovoltaic devices with a power efficiency of 20.1% [10–12]. However, the lack of structural stability of hybrid organic–inorganic QDs [13–17] in comparison with hybrid organic–inorganic perovskite QDs, which exhibit high stability, has limited the applications of hybrid organic–inorganic QDs.

It has been reported that colloidal, all-inorganic (CsPbX_3_, X = Cl, Br, I and their mixture thereof) perovskite QDs possess high luminescence efficiency. Reacting Cs-oleate with a Pb(II)-halide in a high boiling solvent (octadecene) at 140–200 °C, Kovalenko et al. [18] prepared CsPbX_3_ QDs with four edge lengths of 15 nm. These materials exhibited tunable emission wavelength in a range of 410–700 nm, a narrow full width at half maximum (FWHM) of 12–42 nm, and high quantum yield (QY) of 50–90%. In comparison with the synthesis of II–VI and I–III–VI semiconductor QDs, Nedelcu et al. [19] and Akkerman et al. [20] independently demonstrated the feasibility of synthesizing CsPbX_3_ QDs within a few seconds at low temperature. Sun et al. [21] reported the formation of shape-controlled CsPbX_3_ perovskite nanocrystals of three different morphologies via the re-precipitation process at room temperature. Chen et al. [22] prepared cerium lead halide perovskite QDs with a tunable emission peak in the range of 360–700 nm via a non-injection or heating-up method, and the QY of the prepared QDs reached 87%. However, the quality of the QDs synthesized by these methods strongly depends on the concentration gradient, reaction time and temperature, which are difficult to precisely control. These single-batch, time-consuming process methods cannot be used to continuously produce large quantities of QDs.

Microreactor systems have been successfully used to synthesize a variety of QDs [23–24]. With proper selection of chemical reactions and microreactor design, QDs of different characteristics can be synthesized. In addition, microreactor systems with microchannels made from poly(tetrafluoroethylene) (PTFE) capillaries provide a stable environment for the chemical reactions involving air-sensitive materials. Currently, there is no report on the continuous synthesis of perovskite nanocrystals via microreactor systems, even though these systems have been successfully used to synthesize CdSe [25], CdSe/ZnS [26], CdSe*_x_*Te_1−_*_x_* [27], and CdS [28] QDs, as well as monodisperse Au–Ag alloy nanoparticles [29].

In this work, we have constructed a PTFE-based microreactor system in which CsPbX_3_ nanocrystals are continuously synthesized. The fluorescence characteristics of the CsPbX_3_ nanocrystals can be tuned via the changes in the synthesis temperature and the halide composition (Br, I). The photoluminescence (PL) emission spectra of the CsPbX_3_ nanocrystals cover the range of 450–700 nm. The prepared nanocrystals possess excellent optical properties with a FWHM of 19–35 nm and a high fluorescence quantum yield (FLQY) of 47.8–90.55%. The CsPbX_3_ nanocrystals prepared by the PTFE-based microreactor system exhibit three different shapes: spheres, cubes, and rods. This work describes a new route for the continuous synthesis of CsPbX_3_ nanocrystals with luminescence over the whole visible spectral region and demonstrates that inorganic perovskite nanocrystals have potential applications that span a wide gamut from displays to optoelectronic devices.

## Results and Discussion

**Figure 1:**
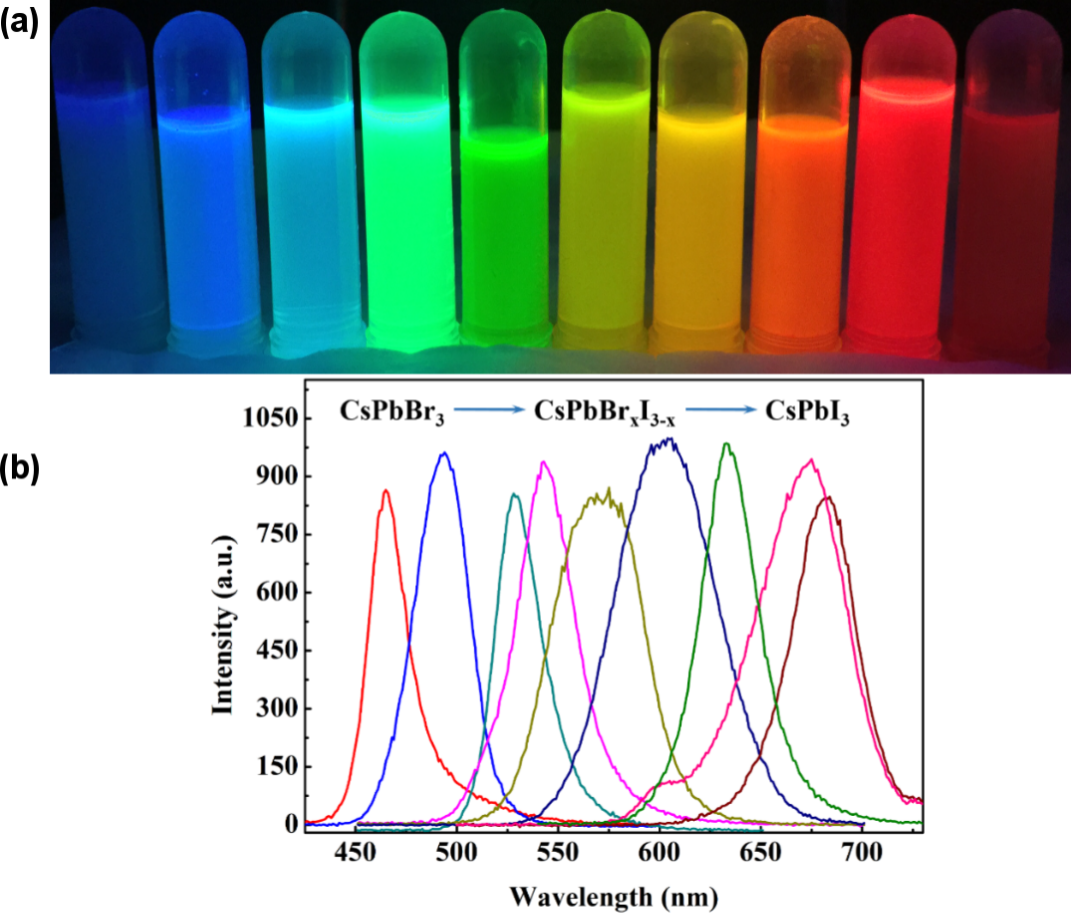
(a) Color luminescence under ultraviolet light and (b) photoluminescence of CsPbX_3_ quantum dot solutions.

Microreactor systems with precise control over the reaction time and temperature offer a possible approach to synthesize high quality QDs in a very short reaction time that emit over the full visible spectrum. This method has distinct advantages over the hot-injection method, which has imprecise control of the reaction time and temperature. These systems make it possible to form CsPbX_3_ QDs (nanocrystals) of high quality by changing the reaction temperature and the molar ratio of the halogen elements. Figure 1 and Figure S1 in Supporting Information File 1 show the color luminescence under irradiation of ultraviolet light and the photoluminescence of the CsPbX_3_ QD solutions made via the PTFE-based microreactor system. The color luminescence covers the full visible spectrum, and the fluorescence spectrum covers the range of 450–700 nm.

**Figure 2:**
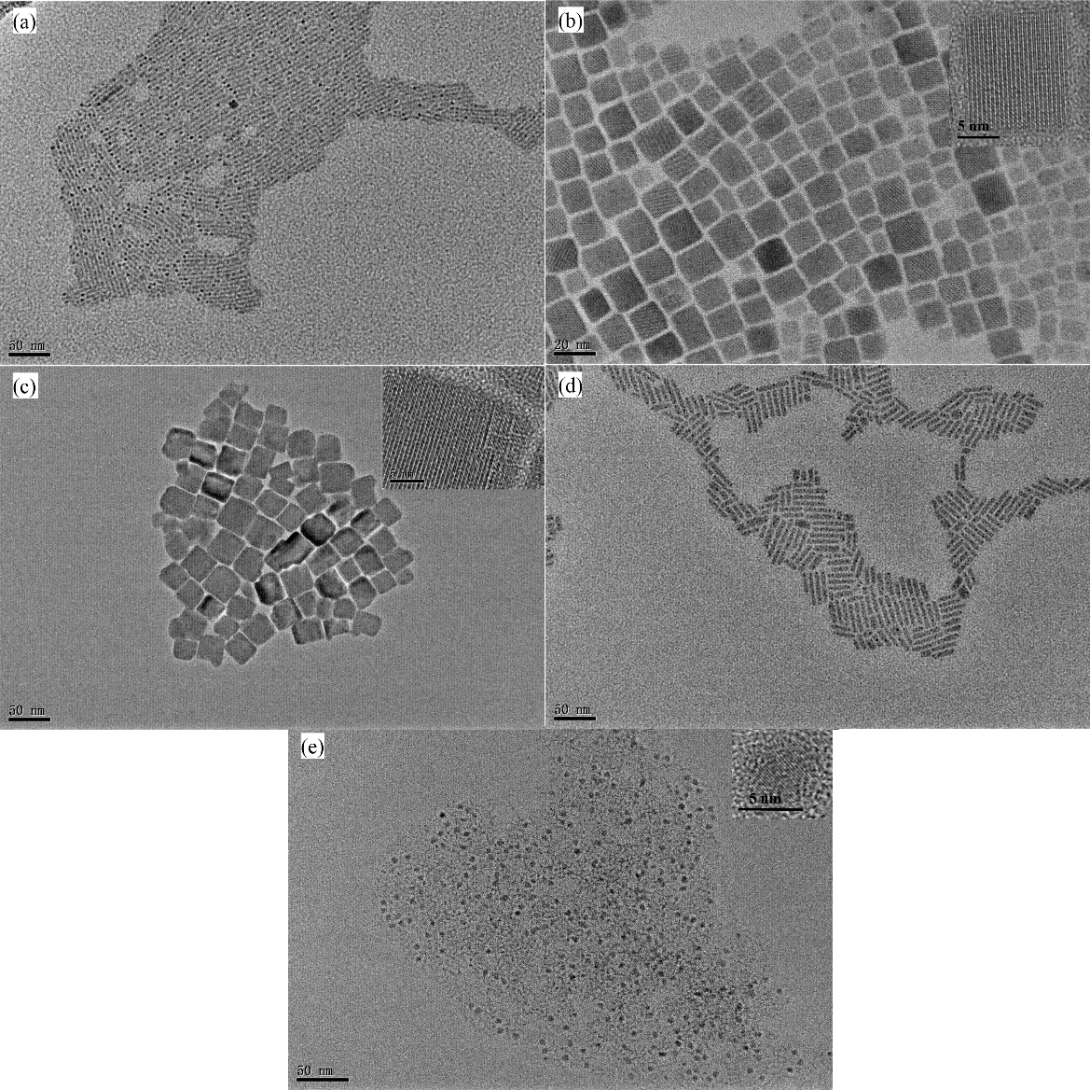
High-resolution transmission electron microscopy images of CsPbX_3_ quantum dots of five different colors: (a) blue, (b) green, (c) yellow, (d) orange, and (e) red.

It is known that colloidal perovskite QDs exhibit an intrinsic quantum size effect. Their size, and morphology, and thus this effect, can be controlled by the reaction temperature, composition and reaction process in the microreactor system. Figure 2 and Figure S2 in Supporting Information File 1 show the transmission electron microscopy (TEM) and high-resolution transmission electron microscopy (HRTEM) (JEM-2100F, JEOL LTD) images of CsPbX_3_ QDs of five different colors. Table S2 in Supporting Information File 1 lists the reaction conditions for the production of the five different perovskite QDs. The blue and yellow QDs (Figure 2a and Figure 2d) have a rod-like shape with a length of ≈50 nm; the green and yellow QDs (Figure 2b and Figure 2c) are cubic shaped with an average size of ≈15 × 35 nm. The red QDs (Figure 2e) are spherical shaped with an average diameter of ≈5 nm and an interplanar distance of 3.23 Å. The difference in the surface morphology is likely due to the orientation dependence of the specific surface energy on the fraction of halogen elements, since the growth orientation of a crystal is controlled by the minimization of total surface energy.

**Figure 3:**
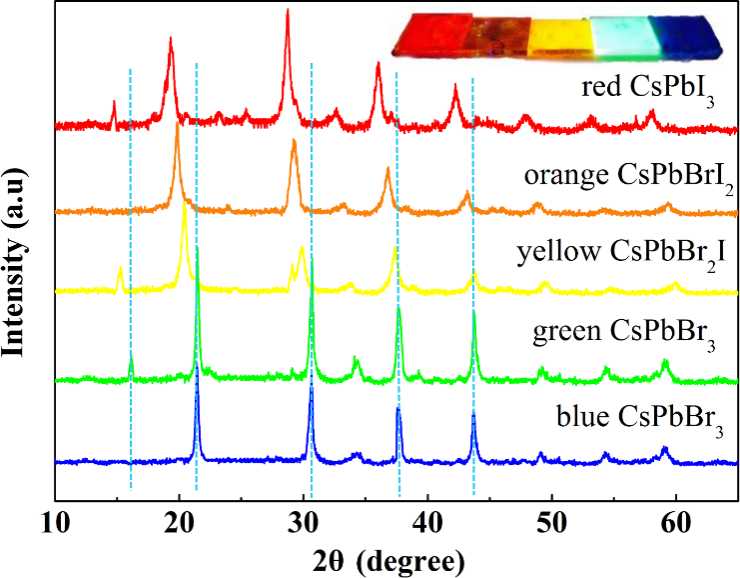
X-ray diffraction patterns of CsPbX_3_ quantum dots of five different colors.

X-ray diffraction (XRD) was used to characterize the structures of the prepared CsPbX_3_ QDs. Figure 3 shows the XRD patterns of the CsPbX_3_ QDs. It is known that there is a small difference in the XRD patterns of the orthorhombic and cubic phases with double peaks at around 2θ = 30° for the orthorhombic phase [30]. One needs to be careful in analyzing the phases of CsPbX_3_ QDs from the XRD patterns. According to Figure 3, there are double peaks at around 2θ = 30° for the yellow CsPbBr_2_I QDs, suggesting that the crystal structure is orthorhombic. For the green CsPbBr_3_ QDs, the peaks located at 2θ values of 16.07°, 21.5°, 30.7°, 34.5°, and 37.6° are associated with the {001}, {110}, {002}, {210} and {211} planes of cubic CsPbBr_3_ (PDF #18-54-0752). There are two crystal structures for the red CsPbI_3_QDs: one is cubic, and the other is orthorhombic. The 2θ values of the peaks for the red CsPbI_3_ QDs shown in Figure 3 are 14.8°, 19.3°, 28.7°, 32.6°, and 36.3° in accord with the results reported in the literature [30–32], confirming that the crystal structure is cubic. The average size of the red perovskite QDs is approximately 4.85 nm as estimated using the fitting of the XRD data, which is compatible with the result of ≈5 nm from the HRTEM results.

Sun et al. [21] pointed out that there exist transitions of the crystal structures of the CsPbI_3_, CsPbI*_x_*Br_3−_*_x_* and CsPbBr_3_ QDs with the increase of bromine, resulting in the shift of the diffraction peak to large angles. Comparing the XRD results of CsPbBr_3_ QDs with CsPbBr_2_I QDs reveals that the addition of iodine causes the reflection shifts of the (110) diffraction peak at 2θ = 20° by ≈1°, and the (002) diffraction peak at 2θ = 30° by ≈0.8°, resulting in the increase of the interplanar distance from 5.66 Å for the CsPbBr_3_ QDs to 6.12 Å for the CsPbBr_2_I QDs. From Figure 3, it is evident that the increasing iodine content causes the shifts of 0.8°, 1.5° and 2° at the diffraction peak at 2θ = 30° for the yellow, orange, and red QDs, respectively, indicating the doping of iodine into the crystal structure. There is no diffraction peak at 2θ = 15° for the blue and orange QDs due to the confinement of the microsystem to the growth of the (001) crystal plane. Note that the TEM images reveal that the blue and orange QDs are present in a rod-like shape. All of these results demonstrate the feasibility of using the PTFE-based microreactor system to synthesize CsPbX_3_ QDs of high quality covering the entire visible spectrum, where QDs of different morphologies can be prepared by controlling the amount and type of halogen element and the reaction temperature.

The reaction temperature plays an important role in the synthesis of the QDs and determines the size and morphology of the QDs. Here, the CsPbBr_3_ QDs were selected to examine the effect of temperature on the growth of the QDs. Figure S5 in Supporting Information File 1 shows the TEM images of the CsPbBr_3_ QDs and the color luminescence of CsPbBr_3_ QD solutions under the excitation of ultraviolet light. The color luminescence of the CsPbBr_3_ QDs formed at the reaction temperatures of 80 °C, 100 °C, 120 °C, and 140 °C exhibits a blue color, and the shape of the CsPbBr_3_ QD changes gradually from a sphere-dominant pattern to a rod-like dominant shape with increasing reaction temperature. The transition temperature for forming CsPbBr_3_ nanocrystals have a mostly rod-like shape is ≈120 °C. For the reaction temperature of 160 °C, the color and shape of the formed CsPbBr_3_ QDs are green and cubic, respectively. Such results demonstrate the morphological dependence of the QDs on the reaction temperature in the PTFE-based microsystem.

UV–vis absorption was used to analyze the optical characteristics of the CsPbX_3_ QD solutions exhibiting a blue, green, yellow, orange and red color (see Figure S3 in Supporting Information File 1). The maximum UV absorption wavelengths are 475 nm (2.61 eV), 523 nm (2.37 eV), 520 nm (2.38 eV), 600 nm (2.07 eV), and 670 nm (1.85 eV) for the blue, green, yellow, orange and red CsPbX_3_ QDs, respectively. The FWHM values of the QDs for the five different colors are very small in the range of 19 to 35 nm, suggesting that the color gamut of the future display made from the CsPbX_3_ QDs will be one and half times of the Nation Television Standard (NTSC) [33].

To assess the luminous efficiency, the quantum yield (QY) of the QDs of five different colors were measured. Table 1 lists the characteristics of the prepared CsPbX_3_ QDs. The CsPbX_3_ QDs have a better QY in the range of 47.8–90.55% (for a standard sample of rhodamine 6G, the QY = 95% in ethanol) than the highly luminescent CdSe QDs reported in literature [25,34–37]. The green QDs have a QY of 90.55%. The QDs with the Stokes shift from 13–35 nm are highly suitable for applications in white-light diodes (WLEDs) [1].

**Table 1:** Characteristics of the CsPbX_3_ quantum dots.

Sample	CsPbX_3_	Color	Emission peak (nm)	Stokes shift (nm)	FWHM^a^ (nm)	QY^b^ (%)

a	CsPbBr_3_	blue	459	15	19	47.80
b	CsPbBr_3_	green	507	19	26	90.55
c	CsPbBr_2_I	yellow	539	35	25	79.48
d	CsPbBrI_2_	orange	576	13	34	71.04
e	CsPbI_3_	red	630	17	35	82.92

^a^Full width at half maximum; ^a^Quantum yield.

**Figure 4:**
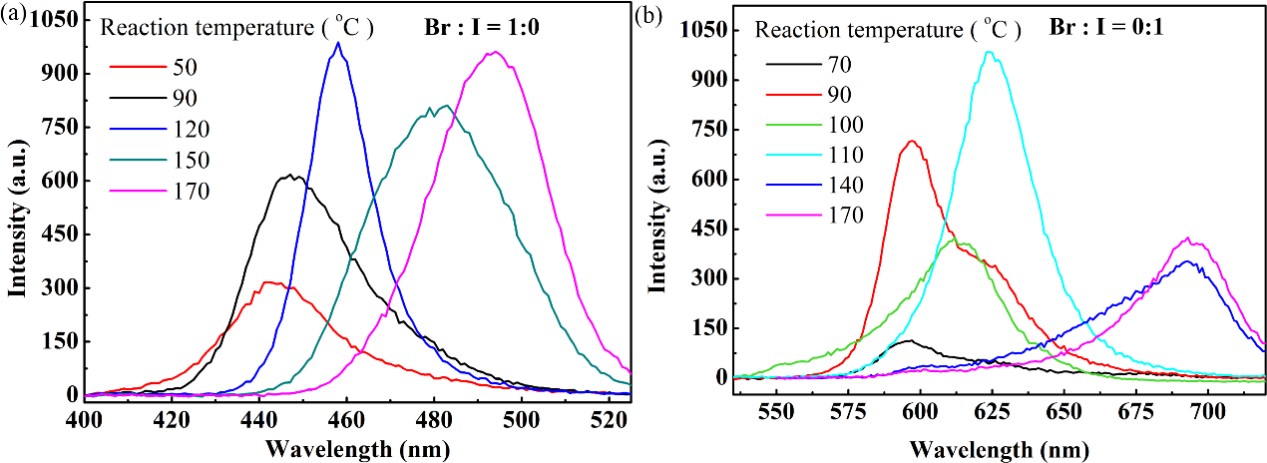
Photoluminescence spectra of CsPbX_3_ quantum dots prepared at different reaction temperatures: (a) X = Br, and (b) X = I.

To study the effect of the reaction temperature on the PL characteristics of the prepared CsPbX_3_ QDs, CsPbX_3_ QDs were synthesized using the PTFE-based microreactor system. The preheat temperature for the synthesis of the CsPbX_3_ QDs was 100 °C, and the temperature of the convection micromixer was in the range of 50–170 °C. The CsPbX_3_ QDs were collected at predetermined temperatures. Figure 4a, Figure S4 and Table S1 in Supporting Information File 1 show the PL spectra of the CsPbBr_3_ QDs prepared at different reaction temperatures. It is evident that the wavelengths corresponding to the PL emission peaks of the CsPbBr_3_ QDs are in the range of 444–495 nm. This result suggests that one can tune the PL characteristics of the CsPbBr_3_ QDs through the control of the reaction temperature in the range of 50–170 °C. From Figure 4a and Figure S4 in Supporting Information File 1, one can note that the wavelengths for the PL emission peaks of the CsPbBr_3_ QDs prepared at the reaction temperatures of 50, 70 and 80 °C are the same. This trend indicates that the critical temperature for the synthesis of CsPbBr_3_ QDs via the PTFE-based microreactor system is 80 °C, and the wavelength of the PL emission peak increases with increasing reaction temperature in the range of 80 to 170 °C.

Using a similar process, the effect of the reaction temperature was studied on the growth of CsPbI_3_ QDs in the PTFE-based microreactor system with the QDs flowing through the microreactor for further growth. The preheat temperature and the prereaction temperature were 100 °C and 70 °C, respectively. Figure 4b, Figure S4 and Table S1 in Supporting Information File 1 depict the PL spectra of the prepared CsPbI_3_ QDs. The wavelengths of the PL emission peaks of the CsPbI_3_ QDs prepared at the reaction temperatures of 70, 80 and 90 °C are the same and there is no observable difference in the PL emission peaks of the CsPbI_3_ QDs prepared at the reaction temperatures in the range of 140–170 °C. The wavelength of the PL emission peak increases from 597 to 695 nm when increasing the temperature of the microreactor from 90 to 170 °C. Such results suggest that one can alter the growth of CsPbI_3_ QDs in the temperature range of 90–170 °C via the PTEF-based microreactor system since the growth of QDs involves the migration/diffusion of atoms/ions. In comparison with the CsPbBr_3_ QDs, the PL spectra of the CsPbI_3_ QDs is much wider, suggesting that the CsPbI_3_ QDs have a smaller band gap than the CsPbBr_3_ QDs [18].

It is known that the wavelength of the PL emission peak of QDs is dependent on the size and composition of the QDs. Alloyed CsPbBr*_x_*I_3−_*_x_* QDs were prepared using the PTFE-based microreactor system. The preheat temperature and the prereaction temperature were 100 °C and 70 °C, respectively. Figure S4 and Table S1 in Supporting Information File 1 show the PL spectra of the alloyed CsPbBr*_x_*I_3−_*_x_* QDs prepared at different reaction temperatures in the range of 70–185 °C for the precursor ratios of Br/I 3:1, 2:1, 1:1, 1:2, and 1:3, respectively.

**Figure 5:**
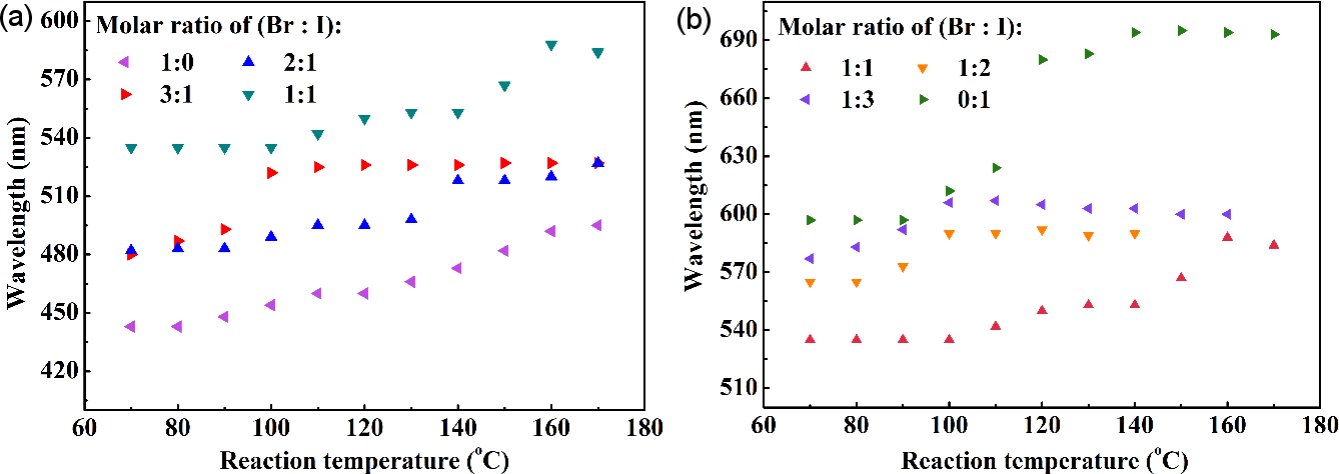
Temperature effect on the photoluminescence peak of the quantum dots prepared with different precursor ratios of Br:I.

From Figure S4 and Table S1 in Supporting Information File 1, one can determine the dependence of the emission wavelength corresponding to the PL peak of the alloyed CsPbBr*_x_*I_3−_*_x_* QDs on the reaction temperature and the precursor ratio of Br/I. Figure 5 shows the variation of the emission wavelength for the PL peak on the reaction temperature for different precursor ratios of Br/I. In general, the emission wavelength for the PL peak increases with increasing reaction temperature, showing a red shift for all the prepared alloyed CsPbBr*_x_*I_3−_*_x_* QDs. For the QDs with the Br/I ratio of 2:1, 3:1, and 1:2, there is a threshold temperature whereby the further increase in the reaction temperature has no effect on the emission wavelength for the corresponding PL peak. Such behavior suggests that both the size and the composition of the QDs are independent of the reaction temperature in the PTFE-based microreactor system, which is likely due to the limited length of the micro-capillaries, i.e., there is not enough time for the further growth of the QDs. It is interesting to note that there is a slight blue shift for the QDs with the Br/I ratio of 1:3 for reaction temperatures larger than 100 °C. The reason for such behavior is unclear; it might be associated with dissociation of ions from the QDs. At high temperatures, ions are in a relatively high energy state, which makes it easy for them to overcome the energy barrier and migrate back to the solution.

Generally, the PL emission wavelength of the QDs is directly related to the energy state of the QDs. The red shift shown in Figure 5 suggests that the band gap of the prepared QDs is temperature dependent, which is similar to the behavior of IV, III–V, and II–VI semiconductors. According to the Varshni empirical relationship, *E*_gap_(*T*) = *E*_gap_(0) − α*T*^2^/(β + *T*) [38], (*E*_gap_(0) is the band gap at 0 K, α is the coefficient of thermal expansion, and β is the Debye temperature), increasing temperature leads to the decease of the band gap. This phenomenon makes it easy for the charge carriers (electrons and/or holes) to be activated and to move to a low energy state in comparison with the charge carriers at low temperatures. The recombination of the charge carriers at a low energy state leads to the emission of light with at a longer wavelength than that at a high energy state, which results in red shift as shown in Figure 5. Such behavior is in accord with the PL behavior of most semiconductors [38–39].

**Figure 6:**
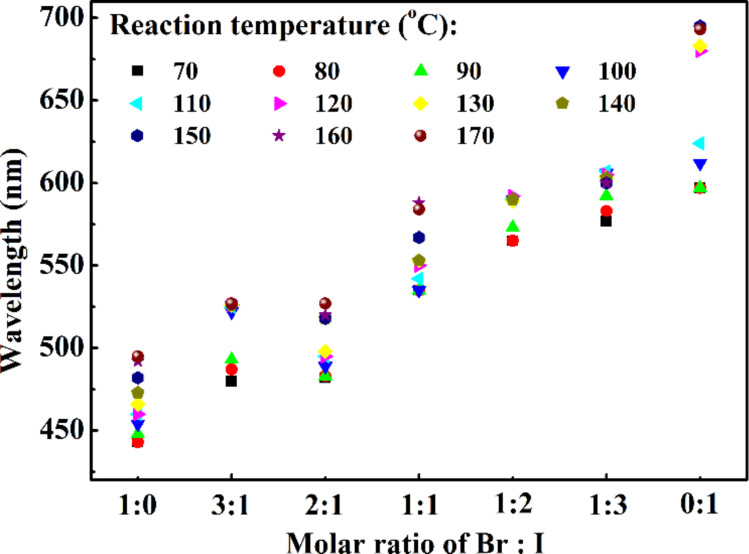
Effect of the Br:I precursor ratio on the peak photoluminescence wavelength of the quantum dots prepared at different process temperatures.

Figure 6 shows the variation of the wavelength of the PL peak of the QDs prepared at different temperatures on the precursor ratio of Br:I. It is evident that increasing the fraction of iodine ions leads to a PL peak at longer wavelengths. With a large portion of Br in the QDs, the wavelength of the PL peak has relatively large, adjustable range through the control of the reaction temperature. In contrast to the large portion of Br in QDs, the QDs prepared with the precursor ratio of Br:I less than 1:1 have a relatively small range of wavelengths that the PL peak can be tuned by the reaction temperature. All of these results demonstrate the effect of chemical composition on the PL characteristics of the prepared QDs. With the control of the chemical composition and reaction temperature, one can tune the PL characteristics of perovskite QDs prepared by the PTFE-based microreactor system.

## Conclusion

In summary, we have constructed a PTFE-based microreactor system to continuously synthesize CsPbX_3_ QDs. The fluorescence emission wavelength of the CsPbX_3_ QDs can be tuned by changing the reaction temperature in the range of 70–180 °C and the Br:I precursor ratio. The PL emission spectra of the prepared CsPbX_3_ QDs cover the extremely wide range of 450–700 nm. The full width at half maximums of the blue, green, yellow, orange and red CsPbX_3_ QDs are very narrow, in the range of 19 to 35 nm. The CsPbX_3_ QDs exhibit good QYs in the range of 47.8–90.55% (the QY of the green QDs was an astounding 90.55%). CsPbX_3_ QDs having spherical, cubic and rod-like shapes were synthesized. Generally, the emission wavelength for the PL peak was found to increase with increasing reaction temperature, exhibiting a red shift for all the prepared alloyed CsPbBr*_x_*I_3−_*_x_* QDs. For the QDs with the Br/I ratios of 2:1, 3:1, and 1:2, there was a threshold temperature at which a further increase in the reaction temperature had no effect on the emission wavelength for the corresponding PL peak. A slight blue shift was found for the QDs with the Br/I ratio of 1:3 for reaction temperatures larger than 100 °C. Increasing the fraction of iodine ions led increase the wavelength of the PL peak of the alloyed CsPbBr*_x_*I_3−_*_x_* QDs.

## Experimental

### Microreactor system

**Figure 7:**
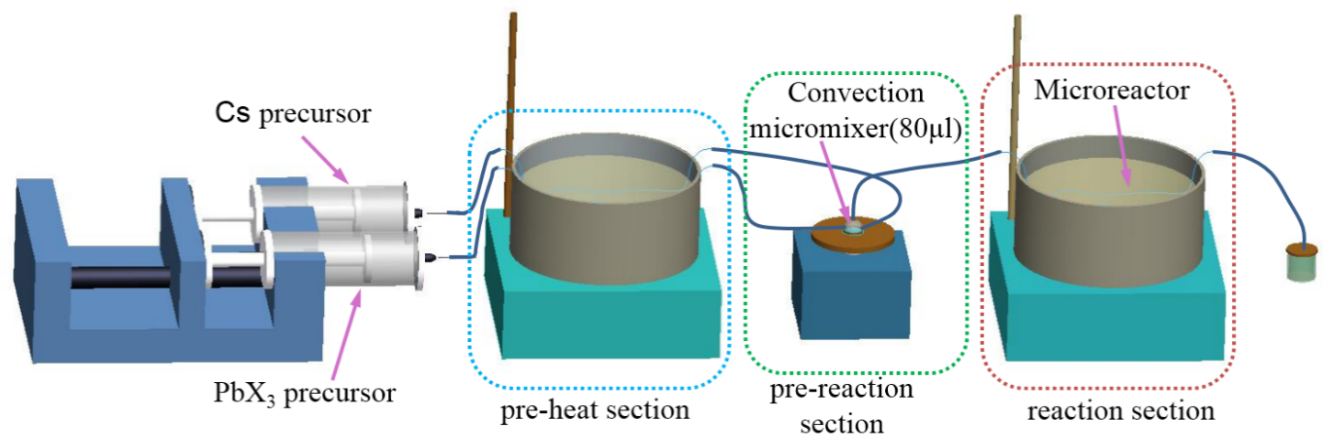
Schematic diagram of a PTFE-based microreactor system.

Figure 7 shows the schematic diagram of the PTFE-based microreactor system. Precursors were rapidly preheated in the preheat segment, which consists of two microchannels of 600 μm inner diameter and a thermally controlled oil bath. A miniature PTFE chamber containing a magnetic stir bar was used for the mixing of two different precursors. The volume of the convection micro-mixer was 80 μL, and the micro-mixer was used to completely mix the precursors for the nucleation of CsPbX_3_ QDs. A three-dimensional serpentine microchannel was constructed by interlacing a PTFE capillary (600 μm inner diameter) along two parallel metal bars (800 μm outer diameter). The PTFE capillaries were used as the microreactor and microchannels. Further nucleation and growth of the CsPbX_3_ QDs occurred in the microreactor, which was immersed in a thermally controlled oil bath. The length of the microreactor and microchannels were 150 cm and 70 cm, respectively.

### Materials

Cesium carbonate (CsCO_3_ 99.9%), lead bromide (PbBr_2_ 99%) and lead II iodide (PbI_2_ 99%) were obtained from Sigma-Aldrich (St. Louis, MO) and used as the precursors. Trioctylphosphine (TOP 90%), oleylamine (OLA 80–90%), 1-octadecene (ODE >90%) and oleic acid (OA, AR) were obtained from Aladdin (Shanghai). All reagents were used as received without any further purification.

### Preparation of Cs precursor solution

0.2 mmol of CsCO_3_, 2.5 mL of OA and 18 mL of ODE were loaded into a three-necked flask of 50 mL, and the mixed solution was magnetically stirred for 1 h at 150 °C in air. The mixed solution of 2 mL was diluted with 24 mL ODE, and the dilute solution was magnetically stirred for 0.5 h until a clear solution was formed.

### Preparation of PbX_2_ precursor solution

The PbX_2_ precursor solution was prepared by heating and magnetically stirring a suspension consisting of 2 mmol PbX_2_ (X = Br, I or Br/I of different ratios), 17 mL ODE, 3 mL OLA, 3 mL OA, and 3 mL TOP at 150 °C for 1 h in air to form a clear solution.

### Synthesis of CsPbX_3_ QDs

Equal volumes of PbX_2_ precursor and Cs precursor solutions, which filled two syringes, respectively, were flowed into the microreactor system at the same flow rate of 10 mL/h. The two precursor solutions were first preheated at a temperature of 100 °C and then mixed in the convection micromixer at a temperature in the range of 50–150 °C. During mixing, CsPbX_3_ QDs quickly nucleated and moved to the reactor for further growth. The formed CsPbX_3_ QDs were collected at the outlet at different reacting temperatures. The reaction temperature was in the range of 70–180 °C.

### Characterization

Absorption spectra were investigated via a UV–vis spectrometer (Varian Cary 50, Varian, Inc.), and the PL spectra were acquired by a Varian Cary Eclipse (Varian, Inc.) spectrophotometer. The quantum yield (QY) of CsPbX_3_ was obtained by comparing the integrated PL intensity of QDs with a standard sample of rhodamine 6G (QY = 95% in ethanol [40]).

HRTEM images were taken on a TEM (JEM-2100F, Jeol, USA) operated at 200 kV, and the sample was prepared by dipping an amorphous carbon–copper grid in a dilute n-hexane dispersed QD solution. The sample was then left to evaporate at room temperature. X-ray diffraction (XRD) measurements were performed on a Rigaku D/max2550 (Rigaku, USA) device operating with Cu Kα (λ = 0.154056 nm), and the QDs were spin-coated on fluorine-doped tin oxide glass.

## Supporting Information

File 1 Additional experimental data, color luminescence, photoluminescence spectra, high-resolution transmission electron microscopy images, absorption and photoluminescence emission spectra.
