# Supplementary material for: PTFE-based microreactor system for the continuous synthesis of full-visible-spectrum emitting cesium lead halide perovskite nanocrystals
Source: Beilstein J Nanotechnol. 2017 Nov 28;8:2521–9. doi: 10.3762/bjnano.8.252 (PMC5727826; doi:10.3762/bjnano.8.252)
Supplement: File 1 — Additional experimental data, color luminescence, photoluminescence spectra, high-resolution transmission electron microscopy images, absorption and photoluminescence emission spectra. [file Beilstein_J_Nanotechnol-08-2521-s001.pdf]

Supporting Information

for

# PTFE-based microreactor system for the continuous synthesis of full-visible-spectrum emitting cesium lead halide perovskite nanocrystals

Chengxi Zhang[1], Weiling Luan[*1],Yuhang Yin[1] and Fuqian Yang[*2]

Address: [1]Key Laboratory of Pressure Systems and Safety (MOE), School of Mechanical and Power Engineering, East China University of Science and Technology, Shanghai 200237, P. R. China, and [2]Department of Chemical and Materials Engineering, University of Kentucky, Lexington, KY 40506, USA

Email: Fuqian Yang - fyang2@uky.edu; Weiling Luan - luan@ecust.edu.cn

* Corresponding author

## Additional experimental data, color luminescence, photoluminescence spectra, high-resolution transmission electron microscopy images, absorption and photoluminescence emission spectra

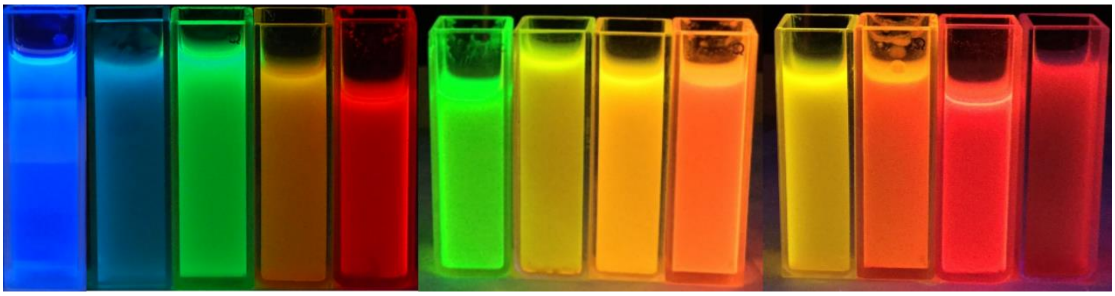

**Figure S1:** Color luminescence of $CsPbX_3$ QDs solutions under ultraviolet light.

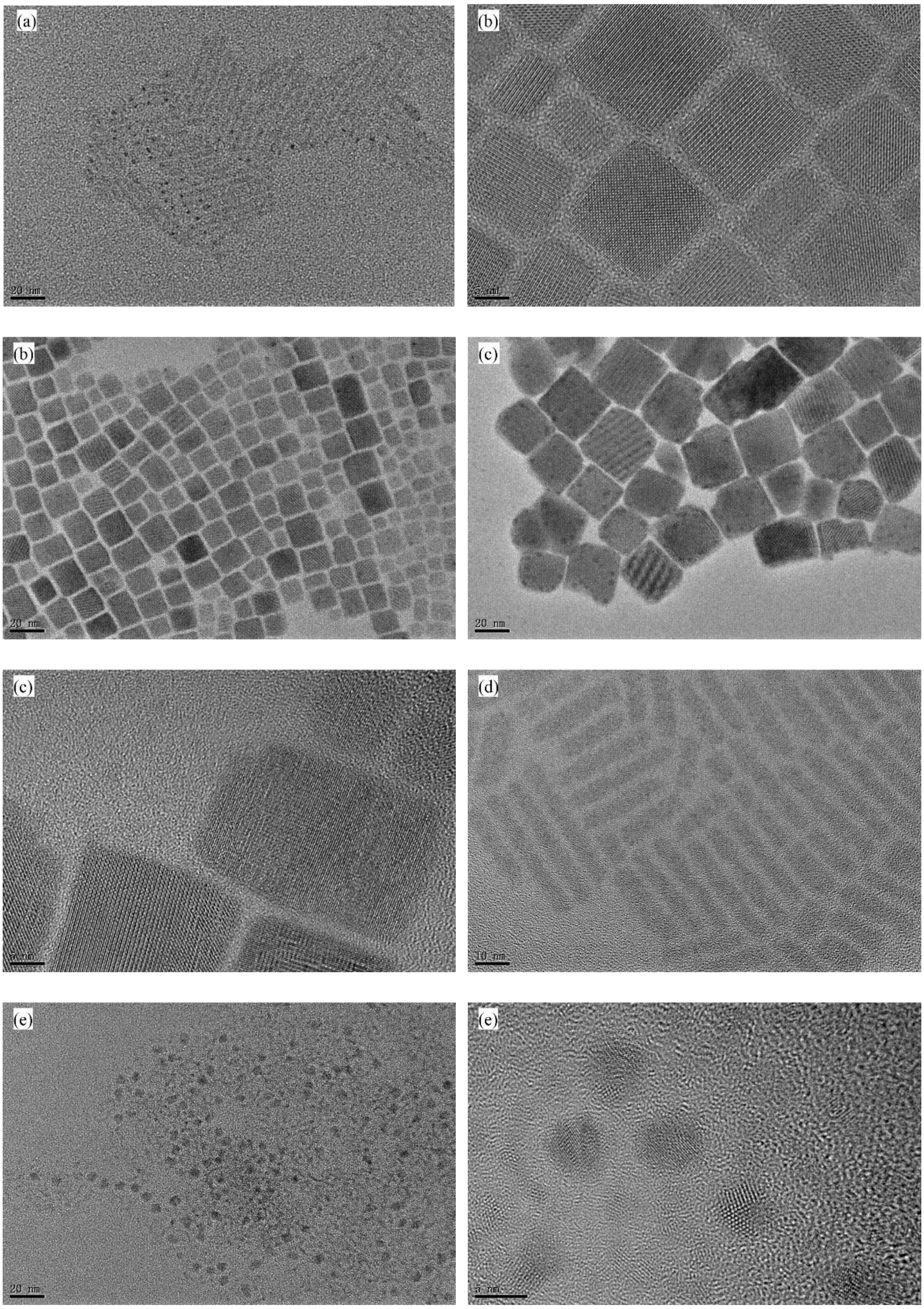


**Figure S2:** HRTEM images of $CsPbX_3$ QDs of five colors; (a) blue, (b) green, (c) yellow, (d) orange, and (e) red.

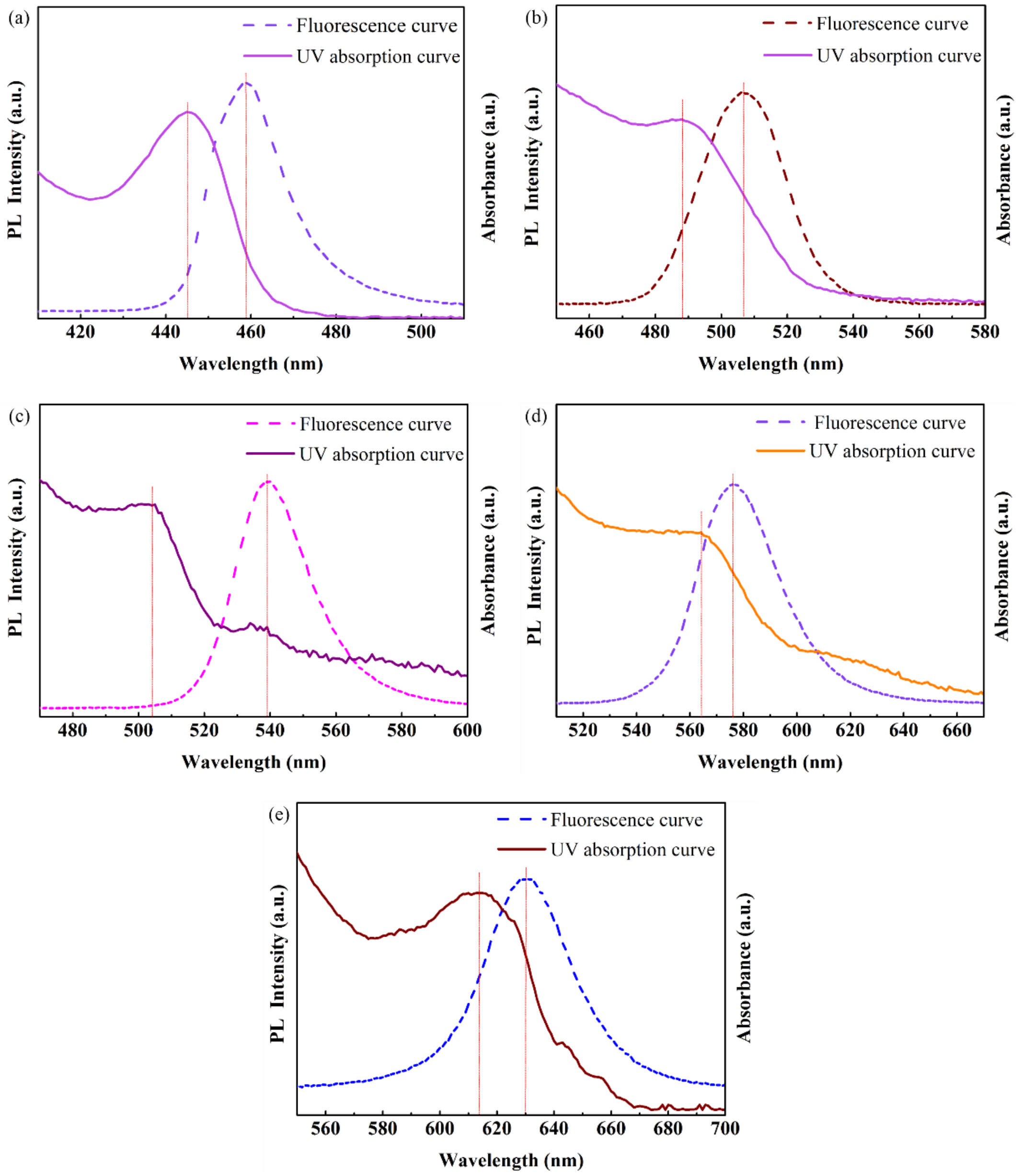


**Figure S3:** Absorption and PL emission spectra of $CsPbX_3$ QDs; (a) blue, (b) green, (c) yellow, (d) orange, and (e) red.

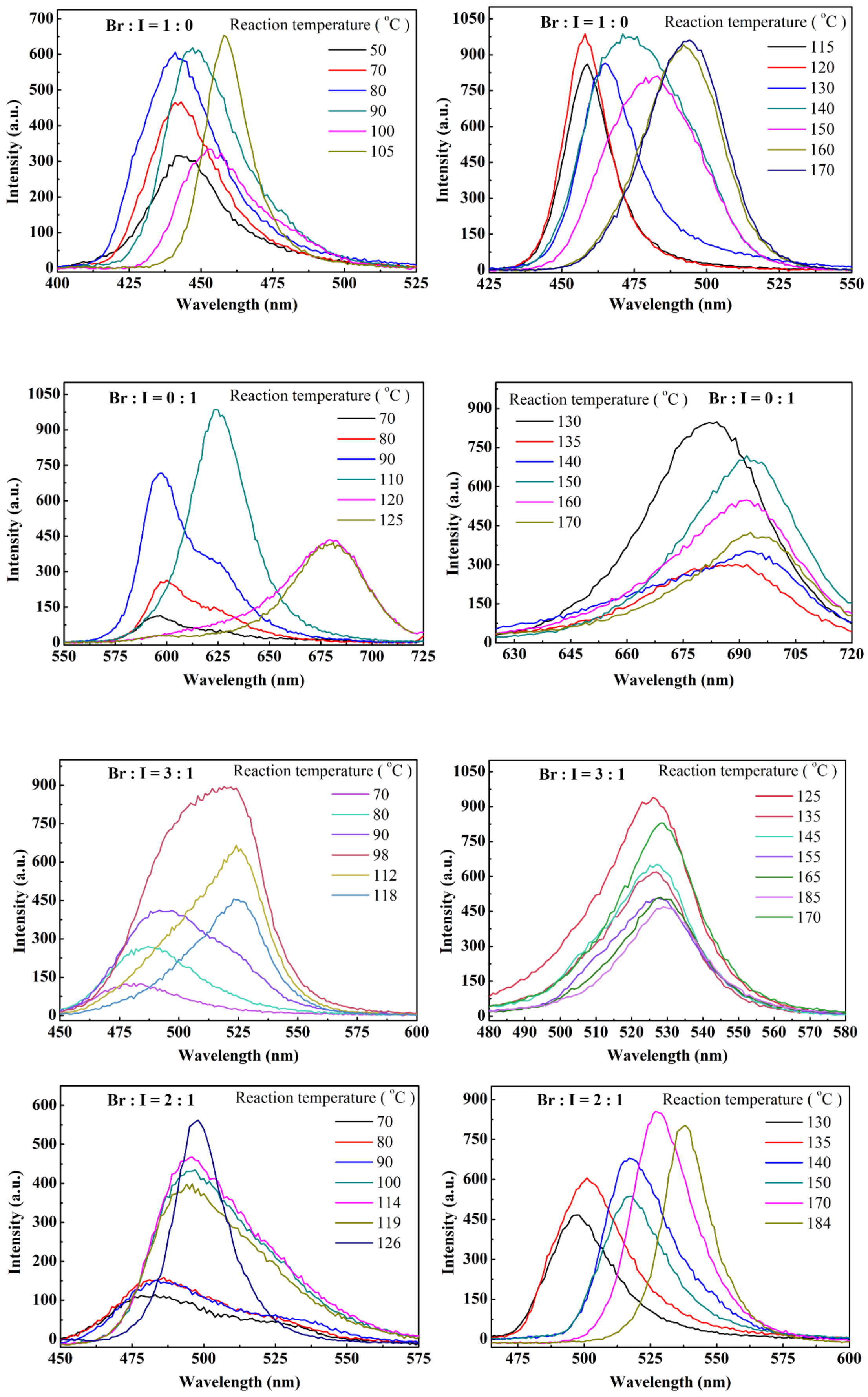
Br : I = 1 : 0
Reaction temperature ( °C )
50
70
80
90
100
105
Br : I = 1 : 0
Reaction temperature ( °C )
115
120
130
140
150
160
170
Br : I = 0 : 1
Reaction temperature ( °C )
70
80
90
110
120
125
Reaction temperature ( °C )
Br : I = 0 : 1
130
135
140
150
160
170
Br : I = 3 : 1
Reaction temperature ( °C )
70
80
90
98
112
118
Br : I = 3 : 1
Reaction temperature ( °C )
125
135
145
155
165
185
170
Br : I = 2 : 1
Reaction temperature ( °C )
70
80
90
100
114
119
126
Br : I = 2 : 1
Reaction temperature ( °C )
130
135
140
150
170
184
Intensity (a.u.)
Wavelength (nm)

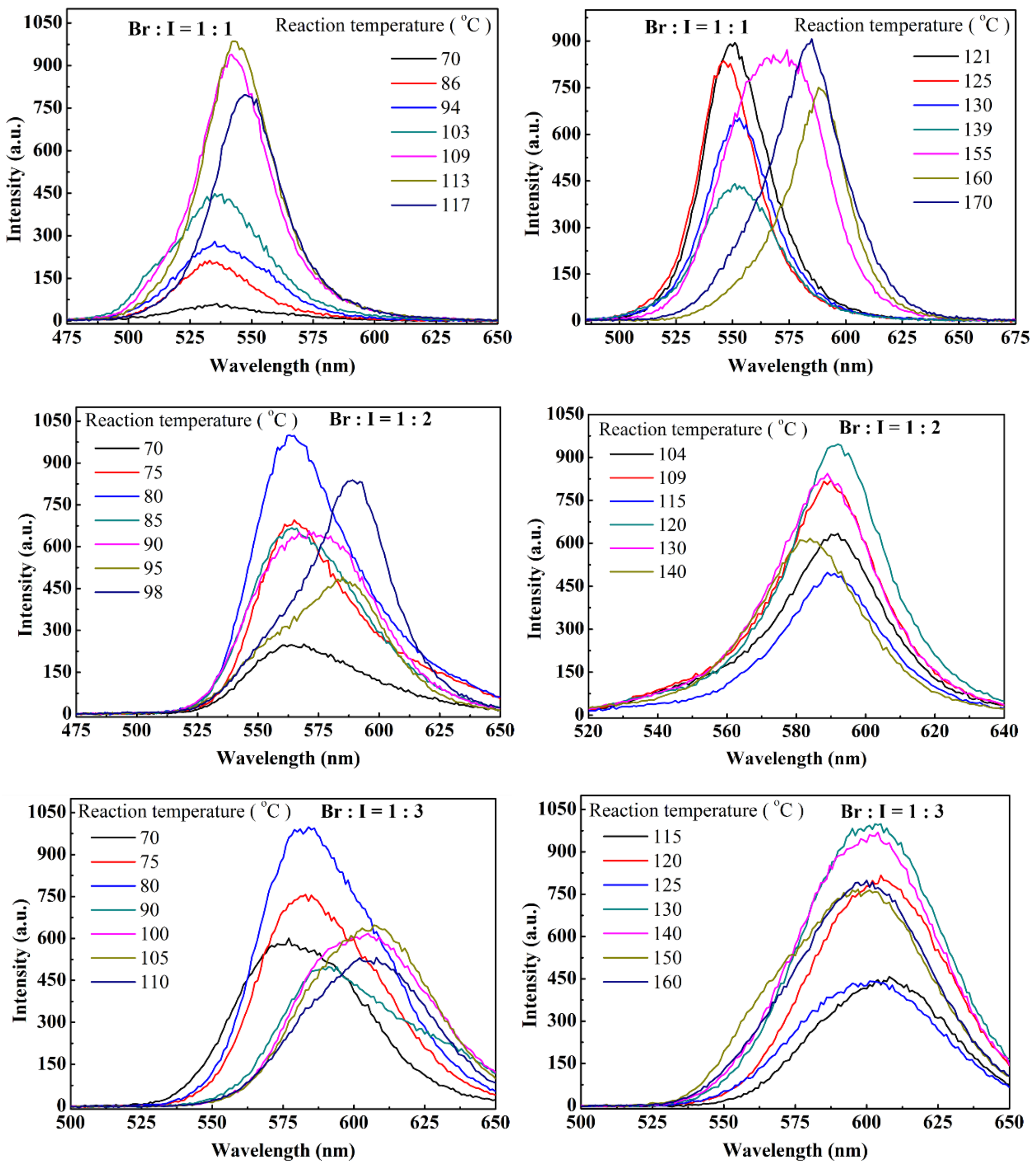


**Figure S4:** PL spectra of $CsPbBr_xI_{3-x}$ QDs synthesized with different mole ratios of Br/I at different reaction temperatures.

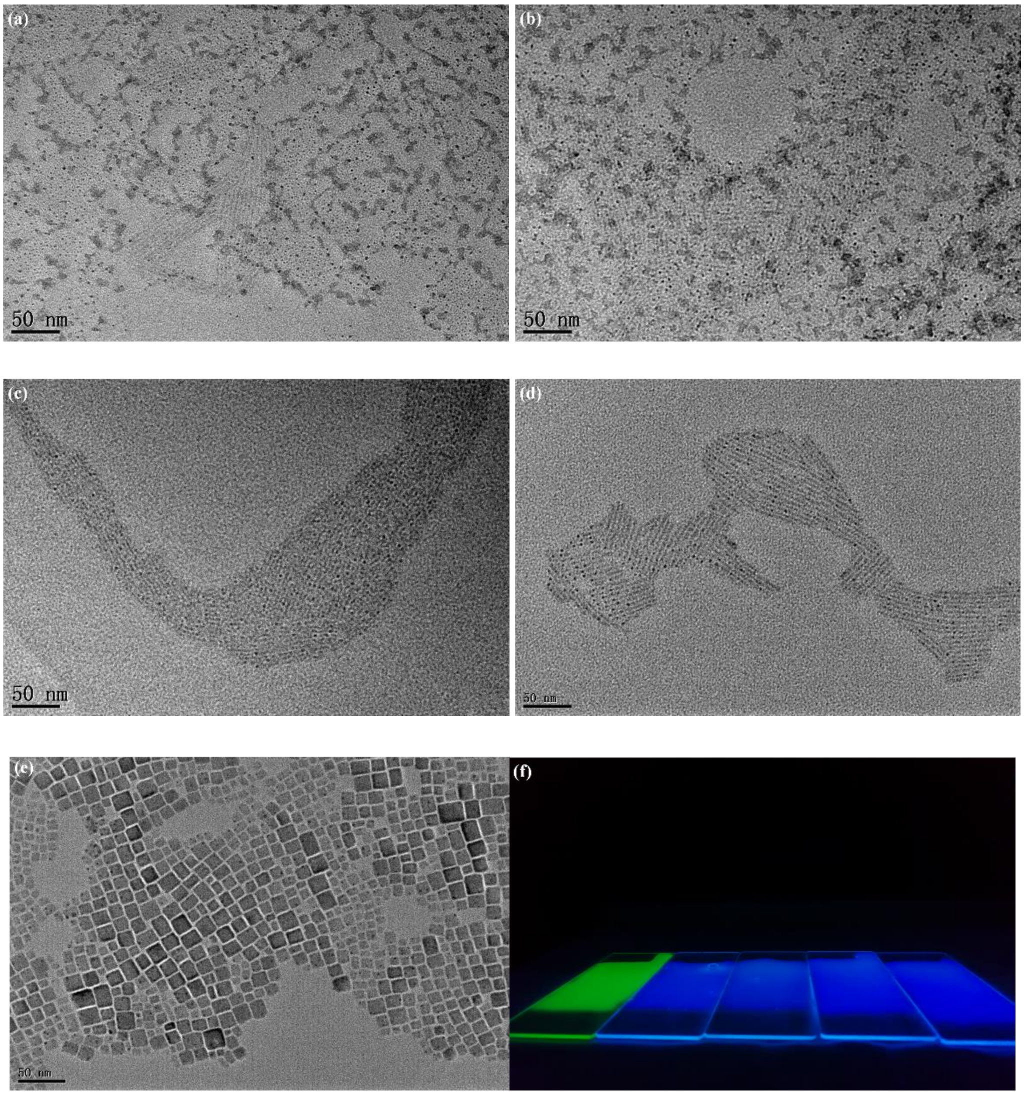


**Figure S5:** HRTEM images of $CsPbBr_3$ QDs synthesized at different reaction temperatures; (a) 80 $^{o}C$, (b) 100 $^{o}C$, (c) 120 $^{o}C$, (d) 140 $^{o}C$, and (e) 160 $^{o}C$; (f) color luminescence of $CsPbBr_3$ QDs solutions under ultraviolet light.

**Table S1:** PL peaks of alloyed $CsPbBr_xI_{3-x}$ QDs prepared at different reaction temperatures.

| Molar ratio Br : I | Reaction temperature (°C) | PL peak (nm) |
|---|---|---|
| 1: 0 | 50 | 444 |
| | 90 | 448 |
| | 120 | 458 |
| | 150 | 483 |
| | 170 | 495 |

| Molar ratio Br : I | Reaction temperature (°C) | PL peak (nm) |
|---|---|---|
| 0 : 1 | 70 | 597 |
| | 90 | 598 |
| | 130 | 682 |
| | 150 | 692 |
| | 170 | 695 |

| Molar ratio Br : I | Reaction temperature (°C) | PL peak (nm) |
|---|---|---|
| 3 : 1 | 70 | 478 |
| | 90 | 492 |
| | 125 | 525 |
| | 155 | 527 |
| | 170 | 528 |

| Molar ratio Br : I | Reaction temperature (°C) | PL peak (nm) |
|---|---|---|
| 2 : 1 | 70 | 481 |
| | 90 | 485 |
| | 119 | 495 |
| | 140 | 517 |
| | 184 | 538 |

| Molar ratio Br : I | Reaction temperature (°C) | PL peak (nm) |
|---|---|---|
| 1 : 1 | 70 | 534 |
| | 94 | 535 |
| | 121 | 551 |
| | 139 | 552 |
| | 170 | 585 |

| Molar ratio Br : I | Reaction temperature (°C) | PL peak (nm) |
|---|---|---|
| 1 : 2 | 70 | 562 |
| | 90 | 571 |
| | 104 | 592 |
| | 120 | 692 |
| | 140 | 582 |

| Molar ratio Br : I | Reaction temperature (°C) | PL peak ( nm ) |
|---|---|---|
| 1 : 3 | 70 | 575 |
| | 90 | 588 |
| | 115 | 608 |
| | 140 | 603 |
| | 160 | 602 |

**Table S2:** Experimental conditions for the synthesis of perovskite QDs.

| | $CsPbX_3$ | Color | Velocity (ml/h) | Pre-reaction temperature ($^oC$) | Reaction length (cm) | Reaction temperature ($^oC$) |
|---|---|---|---|---|---|---|
| a | $CsPbBr_3$ | blue | 10 | 70 | 70 | 120 |
| b | $CsPbBr_3$ | green | 10 | 70 | 70 | 175 |
| c | $CsPbBr_2I$ | yellow | 10 | 70 | 70 | 185 |
| d | $CsPbBrI_2$ | orange | 10 | 70 | 70 | 100 |
| e | $CsPbI_3$ | red | 10 | 70 | 70 | 110 |
